# Supplementary material for: A cell-free nutrient-supplemented perfusate allows four-day ex vivo metabolic preservation of human kidneys
Source: Nat Commun. 2024 May 13;15:3818. doi: 10.1038/s41467-024-47106-w (PMC11091145; doi:10.1038/s41467-024-47106-w)
Supplement: Supplementary file 1 — Supplementary Information [file 41467_2024_47106_MOESM1_ESM.pdf]

LIST OF SUPPLEMENTARY MATERIALS

Supplementary Methods
Supplementary Table 1 | Perfusate composition
Supplementary Table 2 | Donor data of discarded human kidneys.
Supplementary Figure 1 | Temperature-dependence of renal oxygen uptake.
Supplementary Figure 2 | Lipid heterogeneity of human kidney.
Supplementary Figure 3 | Oxidized lipid species in perfusate during 8-day perfusion
Supplementary Figure 4 | Histology after 4-day ex vivo perfusion of human kidneys.
Supplementary Figure 5 | Renal function during 8-day perfusion.
Supplementary Figure 6 | Comparison of perfusion dynamics between porcine and human kidneys.
Supplementary Figure 7 | Macroscopic appearance of porcine kidneys prior to and following auto-transplantation.
Supplementary References

Supplementary Methods

Porcine kidney procurement.

Kidneys from female landrace pigs (±6 months old, ±80 kg) were obtained from a local abattoir. No animal ethical committee approval was required. Pigs were sedated with an electric shock followed by exsanguination. After a warm ischemic period of 20-30 minutes kidneys were obtained and flushed with 100-200 ml of cold Ringers acetate (B. Braun) supplemented with 12.500 IE L^{-1} heparin (LEO Pharma A/S), 10 mg L^{-1} butylscopolaminebromide (Boehringer Ingelheim) and 1 mg L^{-1} nitroglycerine (Hameln). Next, kidneys were flushed with HTK (Histidine-Tryptophan-Ketoglutarate, Custodiol) solution and transported back to the laboratory on ice resulting in a cold ischemic time of 3-6 hours.

Temperature-dependence of renal oxygen uptake.

Porcine kidneys (n=6) were procured as stated above. During back table preparation the renal artery, vein and ureter were cannulated. The artery was flushed with cold DMEM F12, after which the kidney was placed within the organ chamber and connected to a closed-loop perfusion system. A centrifugal pump (Masterflex L/S Digital Drive 600 rpm) perfused the renal artery through silicone tubing (LS25, Masterflex Metrohm) at a mean arterial pressure (MAP) of 75 mmHg. The kidneys were perfused with the same perfusate as described in Table S1. The oxygenator was oxygenated with a carbogen mixture of 95% O_2 and 5% CO_2. Temperature of the perfusion fluid was controlled by a water bath connected to the oxygenator. Arterial and venous partial oxygen pressure (pO_2) were continuously measured using an in-line blood gas sensor (CDI 500 system, Terumo Cardiovascular Systems). Oxygen uptake (mL O_2 min^{-1} 100 gr^{-1}) was calculated as ΔpO_2 (mmHg) x Solubility of O_2 (0.0031 mL O_2 dL $fluid^{-1}$ $mmHg^{-1}$) x Renal Flow (dL min^{-1} 100 gr^{-1}).

The initial temperature of the perfusion solution was set at 20˚C for a minimum of 2 hours after which the perfusate was replaced. Subsequently, baseline perfusion parameters were recorded and temperature was gradually increased. Initially to 25˚C, followed with 3˚C increments every 30 minutes until a temperature of 37 ˚C was reached.

Targeted oxidized lipid analysis

Oxylipids were analyzed from 100 µL perfusate samples according to published protocols[1]. Briefly, to 100 µL perfusate was added 100 µL water, 600 µL methanol and an internal standard mix. Following acidification with formic acid, samples were cleaned up by solid phase extraction using C18 cartridges before being analyzed by LC-MS/MS in MRM mode on a Shimadzu Nexera series UHPLC system coupled to a Sciex 6500 QTrap.

Porcine kidney procurement, perfusion and auto-transplantation

Experiments were performed at the Toronto Organ Preservation Lab (TOPL), following a previously established protocol for porcine kidney procurement and auto-transplantation[2-5]. These experiments were carried out in accordance with the Canadian Council on Animal Care guidelines. Animal ethical approval was granted under an Animal User Protocol (AUP) issued by the University Healthcare Network Animal Care Committee (AUP number 3651).

Male Yorkshire pigs (30-32 kg, 3-months-old) were used. Species-adapted housing with water and food ad libitum was provided. Operative and perioperative procedures, drug administration, and follow-up were conducted as described previously[2,5].

Procurement. Kidney procurement involved anesthesia, intubation, and midline laparotomy. A central venous catheter was placed into the jugular or subclavian vein for fluid administration and blood sampling. Graft procurement included dissection of the right kidney, confirming a single artery and vein, and obtaining sufficient length for cannulation. After resection, the kidney was weighed, the renal artery cannulated, and flushed with 300 mL of histidine-tryptophan-ketoglutarate (HTK) solution at 4°C. Following this minimal warm ischemia (< 5 minutes) and cold ischemia period (< 15 minutes), the kidney was connected to the perfusion platform. Over 4 days, the kidney was perfused within the platform, whilst the pig received daily monitoring, including blood samples, pain medication and antibiotics.

Porcine kidney perfusion. Porcine kidney perfusions followed the same protocol as the human kidneys, with several adjustments. To account for species variations, the porcine kidneys underwent perfusion with an arterial pressure setpoint at 40-65 mmHg, in contrast to the 75 mmHg for the human kidneys. Citric acid was dosed at 5 mmol/L in the substitution solution. Several antioxidants were supplemented in both perfusate and substitution solution (Glycine (2 mmol/L), N-Acetyl-L-Cysteine (2 mmol/L), Taurine (2 mmol/L) and Nicotinamide (1 mmol/L)).

Kidney auto-transplantation. On the fifth day the pigs are re-anesthetized and intubated. An arterial carotid line was placed to allow real-time blood pressure measurements. The original incision was re-opened, and the IVC and abdominal aorta were dissected, and depending on the case, the contralateral kidney was dissected and discarded (Pig/ Kidney#1) or left in situ (Pig/ Kidney#3). Next, the perfused kidney was taken off-pump, weighed, flushed with HTK solution at 4°C, biopsied, and wrapped within a surgical cloth with ice. The kidney was moved to the abdomen, the IVC clamped, systemic heparin administered, and the venous anastomosis made end-to-side. Following IVC unclamping, the abdominal aorta is clamped, an aortomy is performed using a 4-mm aortic punch, and an end-to-side anastomosis is made using the parachute technique. Subsequently, the abdominal aorta is unclamped, and the kidney is reperfused. Systolic blood pressure is maintained above 100 mmHg through titration of norepinephrine infusion. The ureter is reconnected in an end-to-end fashion. Needle biopsies are obtained 30 minutes after reperfusion, the abdominal incision is closed, and blood samples are taken hourly in the first three hours. Once the pig achieves hemodynamic stability without noradrenaline, the arterial line is removed and the pig is transferred to the animal housing, and closely monitored until ambulatory. During post-operative follow-up, the animals receive antibiotics, intravenous fluids, and pain medication as previously described[2,5]. Daily samples are taken for blood gas and biochemical analyses. After seven days, the pig is anesthetized again. Laparotomy is performed, the abdomen inspected, and the kidney graft dissected and sampled, after which the pig is euthanized under anesthesia.

Supplementary Table 1 | Perfusate composition.

**Culture perfusate preparation**				
	**Stock[c]**	**Final[c]**	**Per ±1L**	**Comments**
DMEM F12, HEPES	N/A	N/A	700 mL	Cat# 11330, Gibco.
Human Serum Albumin	200 gr L^{-1}	20 gr L^{-1}	100 mL	Alburex 20, CSL Behring bv.
Insulin-Transferrin-Sodium Selenite (100x)	N/A	N/A	10 mL	Cat# I1884, Sigma-Aldrich. Dissolve 1 vial in 50 mL sterile water.
Sodium bicarbonate	7.5%	N/A	10 mL	Cat# 25080094, Gibco.
Penicillin-streptomycin	5000 U mL^{-1}	50 U mL^{-1}	10 mL	Cat# 15070063, Gibco.
Ciprofloxacin	2 mg mL^{-1}	6 µg mL^{-1}	3 mL	Fresenius Kabi.
Fungizone	0.25 mg mL^{-1}	0.25 µg mL^{-1}	1 mL	Bristol-Myers Squibb.
Citric Acid	1M	5 mM	5 mL	Cat# 3200-1KG, Calbiochem. Merck. Stock solution (1M) made by dissolving Citric Acid into sterile water.
Acetic Acid	2M	2.5 mM	1.25 mL	Cat# 1000631000, EMSURE, Merck. Stock solution (2M) made by Acetic Acid into sterile water.
Sodium Hydroxide	1M	N/A	20-25 mL	Cat# 567530, Calbiochem, Merck. Stock solution (1M) made by dissolving Sodium Hydroxide pellets in sterile water. Add to perfusate until pH range is reached (7.30-7.45).
Sterile water	N/A	N/A	150-200 mL	Sterile water, Versylene Fresenius. Add to perfusate until sodium range is reached (130-145 mmol L^{-1}).
**Substitution perfusate**				
	**Stock[c]**	**Final[c]**	**Per ±1L**	**Comments**
DMEM F12, HEPES	N/A	N/A	700 mL	Cat# 11330, Gibco.
Insulin-Transferrin-Sodium Selenite (100x)	N/A	N/A	10 mL	Cat# I1884, Sigma-Aldrich. Dissolve 1 vial in 50 mL sterile water.
Sodium bicarbonate	7.5%	N/A	10 mL	Cat# 25080094, Gibco.
Penicillin-streptomycin	5000 U mL^{-1}	50 U mL^{-1}	10 mL	Cat# 15070063, Gibco.

Ciprofloxacin	2 mg mL^{-1}	6 µg mL^{-1}	3 mL	Fresenius Kabi.
Fungizone	0.25 mg mL^{-1}	0.25 µg mL^{-1}	1 mL	Bristol-Myers Squibb.
Citric Acid	1M	5 mM	5 mL	Cat# 3200-1KG, Calbiochem. Merck. Stock solution (1M) made by dissolving Citric Acid into sterile water.
Acetic Acid	2M	2.5 mM	1.25 mL	Cat# 1000631000, EMSURE, Merck. Stock solution (2M) made by Acetic Acid into sterile water.
Sodium Hydroxide	1M	N/A	20-25 mL	Cat# 567530, Calbiochem, Merck. Stock solution (1M) made by dissolving Sodium Hydroxide pellets in sterile water. Add to perfusate until pH range is reached (7.30-7.45).
Sterile water	N/A	N/A	150-200 mL	Sterile water, Versylene Fresenius. Add to perfusate until sodium range is reached (130-145 mmol L^{-1}).

Supplementary Table 2 | Donor data of discarded human kidneys.

	**8-day Organ Culture**			**4-day Organ Culture**				
	**Day8_1**	**Day8_2**	**Day8_3**	**Day4_1**	**Day4_2**	**Day4_3**	**Day4_4**	**Day4_5**
**Donor age (Y)**	50-59	50-59	70-79	60-69	70-79	70-79	50-59	40-49
**Gender (M/F)**	M	M	M	M	F	M	F	M
**BMI**	30	30	25	24	27	25	16	16
**Hypertension**	N	N	Y	N	Y	Y	N	Y
**Diabetes Mellitus**	N	N	Y	Y	N	Y	N	Y
**Smoking**	Y	Y	Y	N	N	Y	Y	Y
**Cause of death**	Cardiac arrest	Cardiac arrest	Cardiac arrest	CVA	SAH	CVA	Euthanasia	CVA
**Donor type**	DBD	DBD	DCD	DCD	DBD	DBD	DCD	DBD
**ICU stay (days)**	1	1	3	2	1	1	3	2
**Peak LDH (@ICU) (U/L)**	943	943	760	239	684	282	196	206
**Peak serum creatinine (@ICU) (µmol/L)**	257	257	96	89	102	81	51	102
**Diuresis (@ICU) (ml/hr)[1]**	35	35	250	75	100	100	33	120
**EGFR**	UNKN	UNKN	82	83	82	95	122	99

**Urine sediment protein**	0.96 g L^{-1}	0.96 g L^{-1}	0.48 g L^{-1}	0.36 g L^{-1}	< 0.20 g L^{-1}	< 0.20 g L^{-1}	< 0.20 g L^{-1}	1.6 g L^{-1}
**Reason of discard**	Renal dysfunction in medical history	Renal dysfunction in medical history	Inability to allocate	Medical reasons	Medical reasons	Suspected malignancy	Inability to allocate	Previously transplanted organ
**Transportation**	SCS	SCS	NRP; SCS.	SCS	SCS	SCS	HMP	SCS

Abbreviations: BMI, Body Mass Index; CVA, Cerebral Vascular Accident; SAH, Subarachnoid Hemorrhage; DBD, Donation after Brain Death; DCD, Donation after Cardiac Death; ICU, Intensive Care Unit; LDH, Lactate dehydrogenase; EGFR, Estimated Glomerular Filtration Function; DM, Diabetes Mellitus; SCS, Static Cold Storage; NRP, Normothermic Regional Perfusion; HMP, Hypothermic Machine Perfusion.

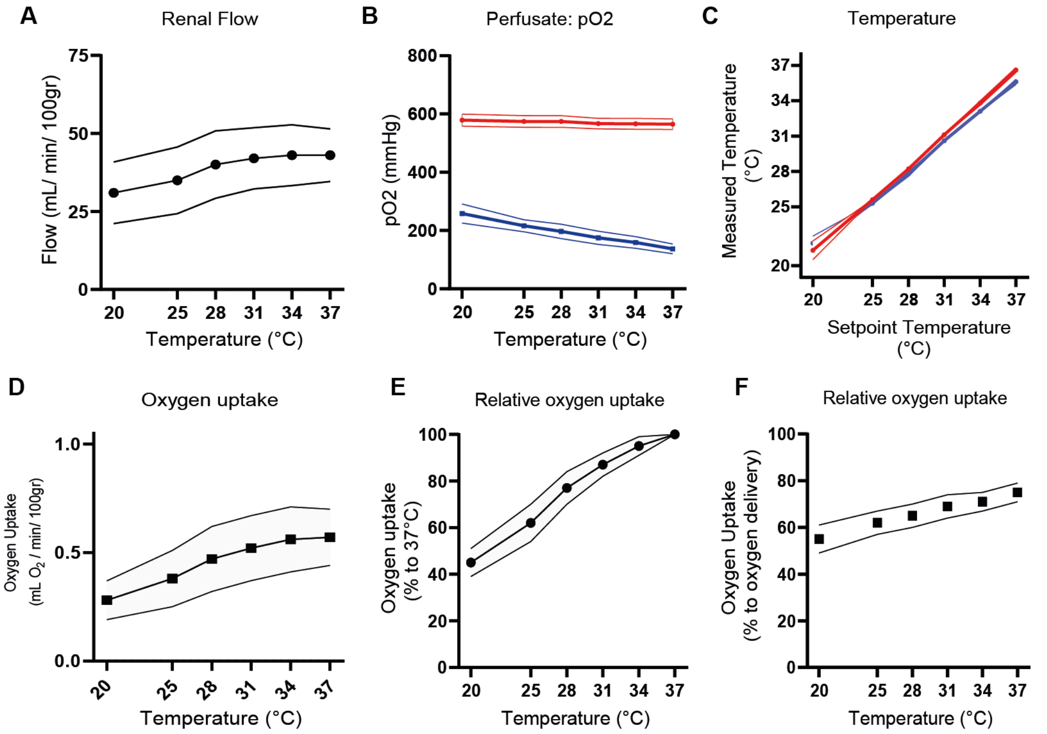

Supplementary Figure 1 | Temperature-dependence of renal oxygen uptake. Temperature determines metabolic rate and therewith oxygen uptake. The effect of temperature on whole kidney oxygen uptake was determined in a closed perfusion model using porcine kidneys (n=6) that were perfused with an acellular perfusate whilst perfusion temperature was gradually increased. **A,** Renal flow during kidney perfusion at different temperatures. **B,** Arterial and venous partial oxygen pressure during kidney perfusion at different temperatures. **C,** Measured temperature during kidney perfusion at different setpoint temperatures. **D,** Oxygen uptake at different temperatures. **E,** Oxygen uptake relative to uptake at 37°C during kidney perfusion. **F,** Oxygen uptake relative to arterial oxygen delivery showing the percentage of available oxygen that is consumed during kidney perfusion at different temperatures. Data are presented as mean±SEM. Source data are provided as a Source Data file.

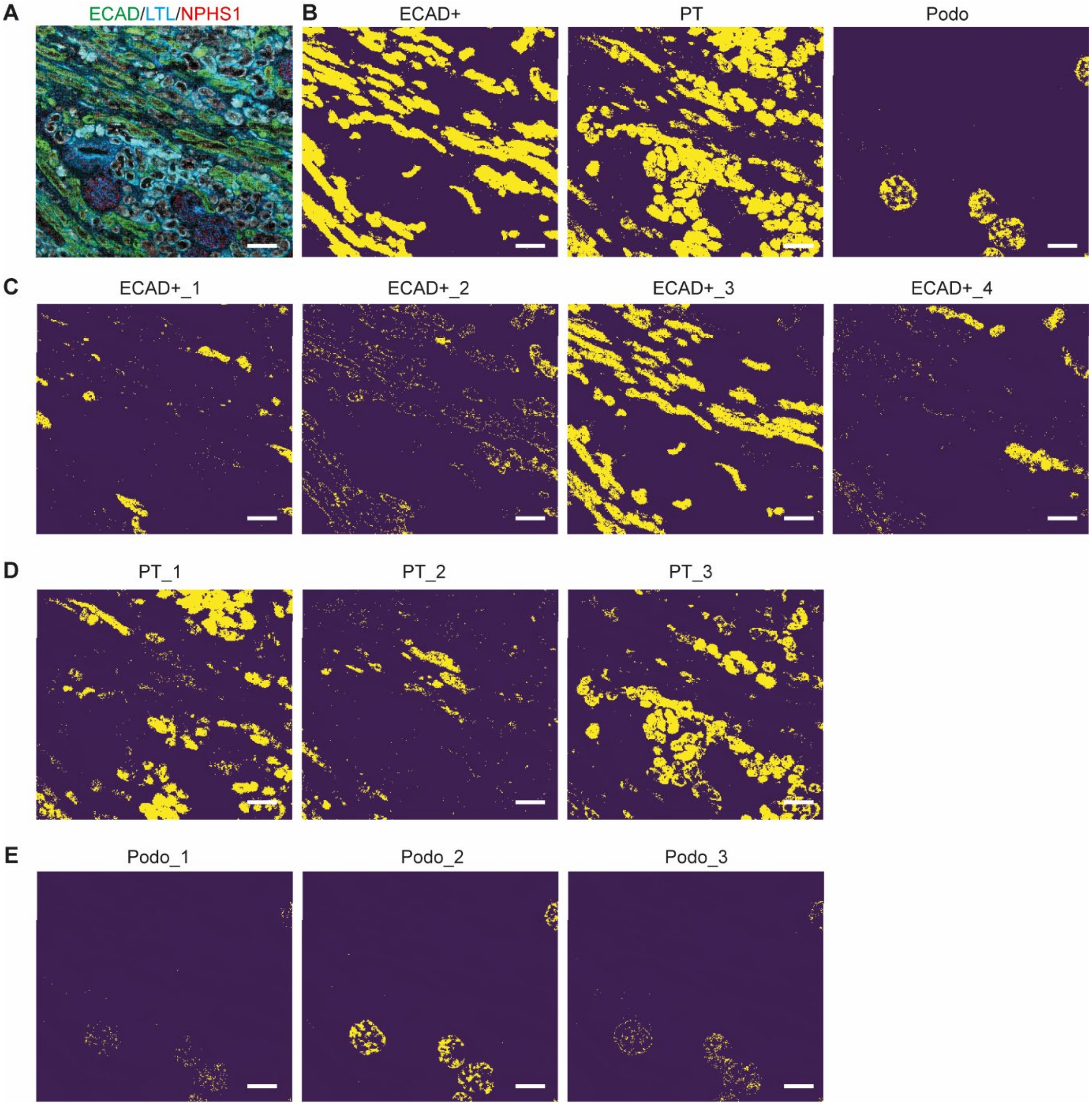

Supplementary Figure 2 | Lipid heterogeneity of human kidney. A, Immunofluorescence staining (LTL, E-cadherin (ECAD) and NPHS1) on post-MALDI-MSI tissue obtained at Day8 of the 8-day organ culture period. **B,** Distribution of different epithelial cell clusters on tissue as identified in Figure 3A. **C-E,** Distribution of different phenotype of epithelial cells on tissue as identified in Figure 3B-D. Bars represent 200 µm.

A

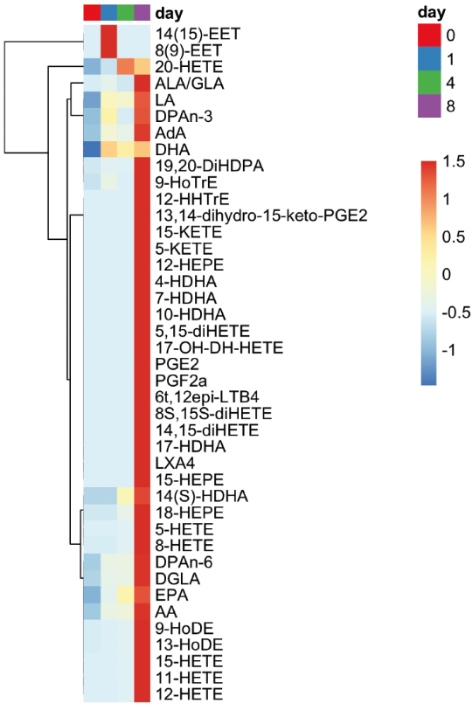

Supplementary Figure 3 | Oxidized lipid species in perfusate during 8-day perfusion. **A**, Heatmap visualization of relative fold change in oxidized lipid species in the perfusate of Kidney Day8_2 during 8-day perfusion, as measured by targeted lipidomics of oxylipids. Source data are provided as a Source Data file.

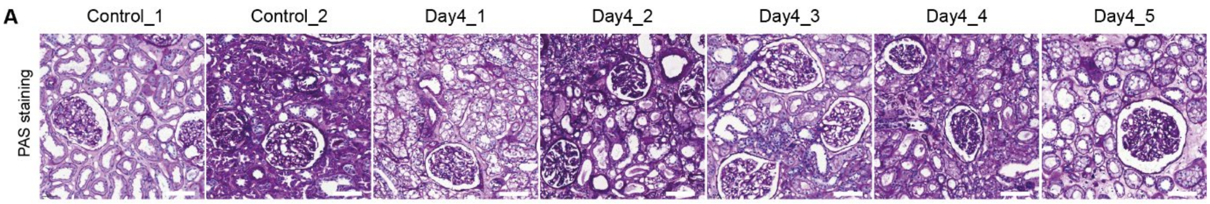

Supplementary Figure 4 | Histology after 4-day ex vivo perfusion of human kidneys. A, Representative Periodic Acid-Schiff (PAS) staining for the five human kidneys that were perfused for the 4-day period. Day4_1 and Day4_2 are the contralateral kidneys of Control_1 and Control_2, respectively. Bars represent 100 µm.

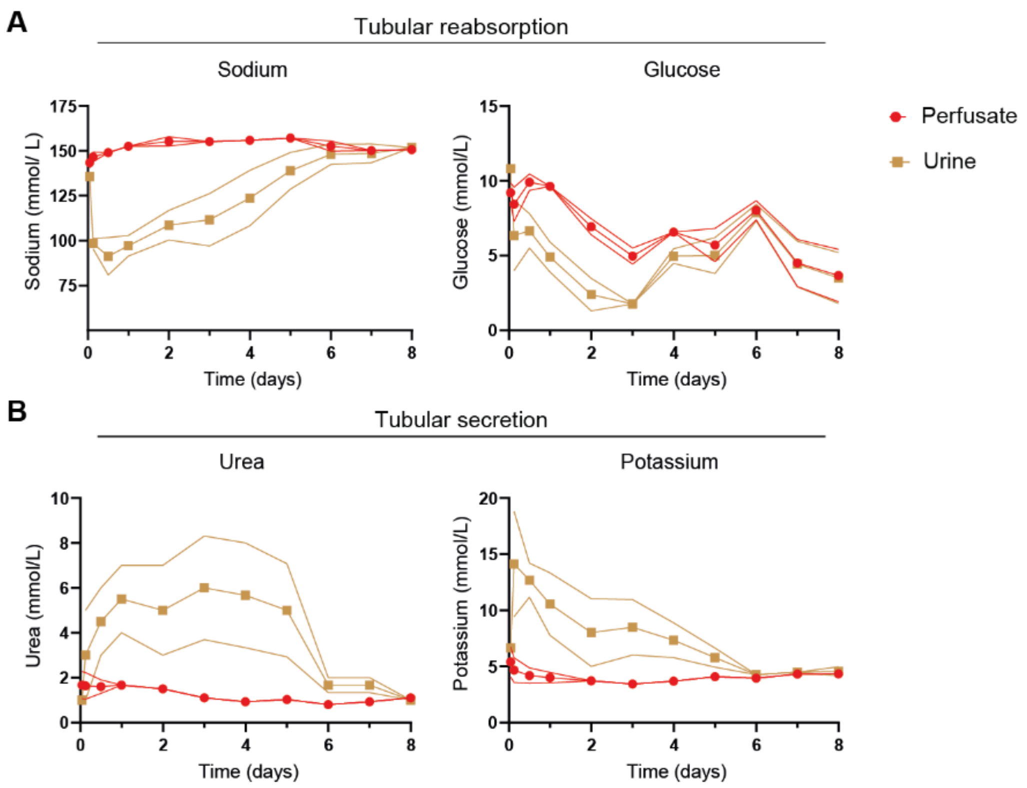

Supplementary Figure 5 | Renal function during 8-day perfusion. Renal function during the 8-day subnormothermic culture of three discarded human kidneys. Concentration gradients between perfusate and urine were maintained until Day4-Day6 of perfusion. **A**, Perfusate and urine concentration of sodium and glucose demonstrates tubular reabsorption**. B**, Perfusate and urine concentration of urea and potassium demonstrates tubular secretion. Data are presented as mean±SEM. Source data are provided as a Source Data file.

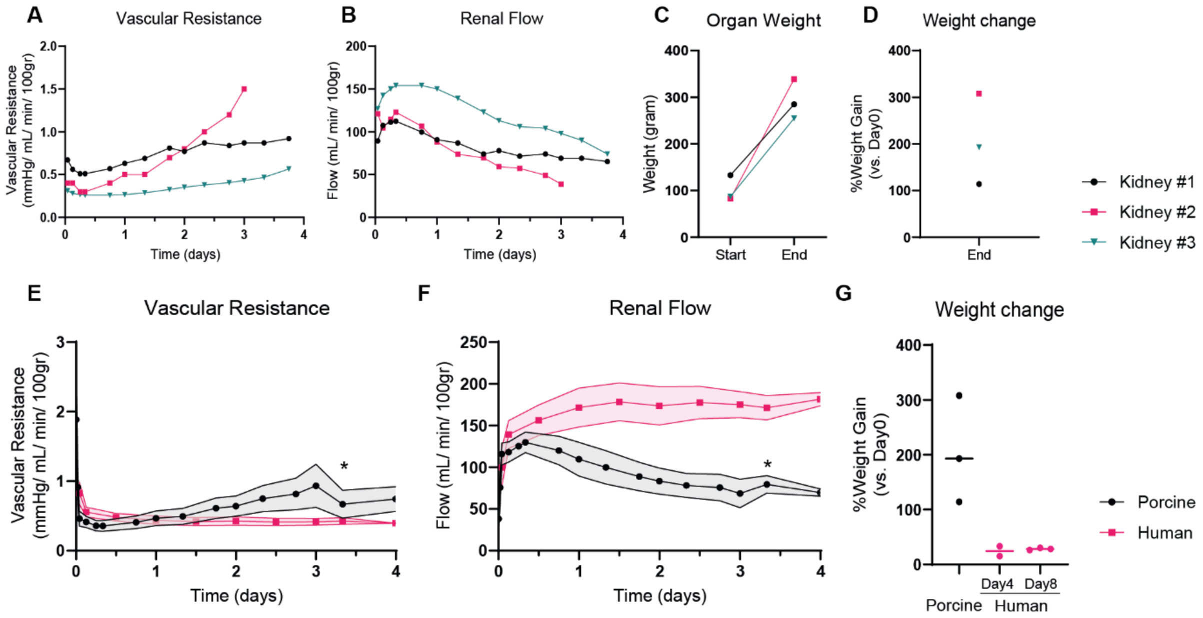

Supplementary Figure 6 | Comparison of perfusion dynamics between porcine and human kidneys. Porcine kidneys were procured from male Yorkshire pigs (30 kg, 3-month-old) following minimal warm ischemia in a living-donor setting. **A**, Vascular resistance. **B**, Renal flow. **C**, Whole organ weight at start and end of perfusion. **D**, Weight change during perfusion. **E**, Vascular resistance dynamics in porcine and human kidneys (n=3 per group). **F**, Renal flow dynamics in porcine and human kidneys. For E and F * denotes the termination of porcine kidney perfusion #2 (n=3 per group). **G**, Weight change during porcine and human kidney perfusion. Source data are provided as a Source Data file.

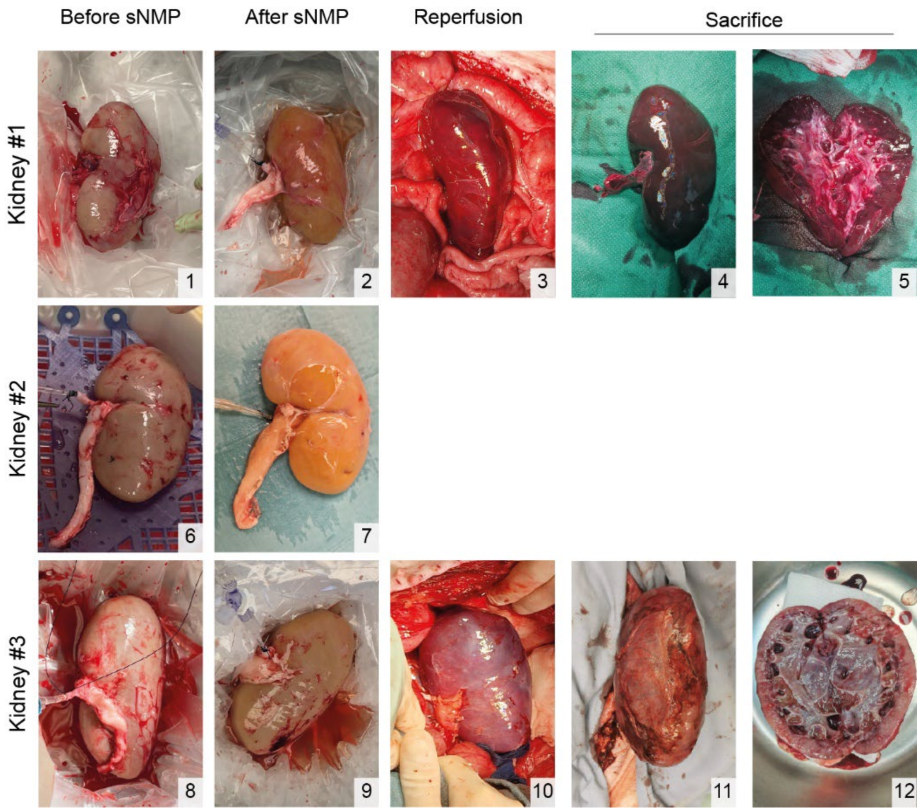

Supplementary Figure 7 | Macroscopic appearance of porcine kidneys prior to and following auto-transplantation. Macroscopic appearance of the porcine kidneys from Figure S6 after procurement prior to starting sNMP (1,6,8), at the end of sNMP (2,7,9), approximately 30 minutes after reperfusion (3, 10) and at sacrifice (4,5,11,12).

Supplementary References

1. Körner A, Zhou E, Müller C, Mohammed Y, Herceg S, Bracher F, et al. Inhibition of Δ24-dehydrocholesterol reductase activates pro-resolving lipid mediator biosynthesis and inflammation resolution. Proc Natl Acad Sci U S A. 2019;116(41):20623-34.
2. Kaths JM, Echeverri J, Goldaracena N, Louis KS, Yip P, John R, et al. Heterotopic Renal Autotransplantation in a Porcine Model: A Step-by-Step Protocol. J Vis Exp. 2016(108):53765.
3. Kaths JM, Cen JY, Chun YM, Echeverri J, Linares I, Ganesh S, et al. Continuous Normothermic Ex Vivo Kidney Perfusion Is Superior to Brief Normothermic Perfusion Following Static Cold Storage in Donation After Circulatory Death Pig Kidney Transplantation. Am J Transplant. 2017;17(4):957-69.
4. Kaths JM, Hamar M, Echeverri J, Linares I, Urbanellis P, Cen JY, et al. Normothermic ex vivo kidney perfusion for graft quality assessment prior to transplantation. Am J Transplant. 2018;18(3):580-9.
5. Parmentier C, Gao F, Ray S, Kawamura M, Noguiera E, Ganesh S, et al. Intubation, Central Venous Catheter, and Arterial Line Placement in Swine for Translational Research in Abdominal Transplantation Surgery. J Vis Exp. 2023(192).
