# Supplementary material for: A cell-free nutrient-supplemented perfusate allows four-day ex vivo metabolic preservation of human kidneys
Source: Nat Commun. 2024 May 13;15:3818. doi: 10.1038/s41467-024-47106-w (PMC11091145; doi:10.1038/s41467-024-47106-w)
Supplement: Supplementary file 3 — Reporting Summary [file 41467_2024_47106_MOESM3_ESM.pdf]

nature portfolio

Corresponding author(s): Ton Rabelink

Last updated by author(s): Mar 18, 2024

Reporting Summary

Nature Portfolio wishes to improve the reproducibility of the work that we publish. This form provides structure for consistency and transparency in reporting. For further information on Nature Portfolio policies, see our Editorial Policies and the Editorial Policy Checklist.

Statistics

For all statistical analyses, confirm that the following items are present in the figure legend, table legend, main text, or Methods section.

n/a	Confirmed	
☐	☒	The exact sample size (n) for each experimental group/condition, given as a discrete number and unit of measurement
☐	☒	A statement on whether measurements were taken from distinct samples or whether the same sample was measured repeatedly
☐	☒	The statistical test(s) used AND whether they are one- or two-sided<br>*Only common tests should be described solely by name; describe more complex techniques in the Methods section.*
☒	☐	A description of all covariates tested
☐	☒	A description of any assumptions or corrections, such as tests of normality and adjustment for multiple comparisons
☐	☒	A full description of the statistical parameters including central tendency (e.g. means) or other basic estimates (e.g. regression coefficient) AND variation (e.g. standard deviation) or associated estimates of uncertainty (e.g. confidence intervals)
☐	☒	For null hypothesis testing, the test statistic (e.g. F, t, r) with confidence intervals, effect sizes, degrees of freedom and P value noted<br>*Give P values as exact values whenever suitable.*
☒	☐	For Bayesian analysis, information on the choice of priors and Markov chain Monte Carlo settings
☒	☐	For hierarchical and complex designs, identification of the appropriate level for tests and full reporting of outcomes
☒	☐	Estimates of effect sizes (e.g. Cohen's d, Pearson's r), indicating how they were calculated

Our web collection on statistics for biologists contains articles on many of the points above.

Software and code

Policy information about availability of computer code

Data collection	flexControl (Version 4.0, Bruker Daltonics); Sciex Analyst (version 1.8.1, Sciex);
Data analysis	flexImaging 5.0 (Bruker Daltonics); SCiLS Lab 2016b (version 2016b, Bruker Daltonics); CaseViewer (version 2.4, 3DHISTECH Ltd); R (version 4.0); Rstudio (version 1.4.1717); Matlab (version R2019a; Mathworks); Seurat 3.0; IsoCorrectoR (version 1.14.0); MS-DIAL (version 5.1); Sciex OS (Sciex); Graphpad Prism (version 9.3.1).<br>The code used in this study has been published previously after peer-review and is available at https://doi.org/10.5281/zenodo.7191331

For manuscripts utilizing custom algorithms or software that are central to the research but not yet described in published literature, software must be made available to editors and reviewers. We strongly encourage code deposition in a community repository (e.g. GitHub). See the Nature Portfolio guidelines for submitting code & software for further information.

Data

Policy information about availability of data

All manuscripts must include a data availability statement. This statement should provide the following information, where applicable:
- Accession codes, unique identifiers, or web links for publicly available datasets
- A description of any restrictions on data availability
- For clinical datasets or third party data, please ensure that the statement adheres to our policy

All the exported and processed MALDI-MSI data underlying the main text and supplementary materials were deposited in Figshare at https://doi.org/10.6084/

m9.figshare.25304326. Owing to the large size of the raw MALDI-MSI data this could not be deposited in a public repository. The full MALDI imaging data generated in this study are available upon request from the corresponding author (please contact Gangqi Wang and Ton Rabelink).
The Human Metabolome Database (https://hmdb.ca/) was used for lipid and metabolite annotation (MSI data analysis).
The MS-DIAL lipid database version Msp20221205132019 was used for lipid annotation (untargeted lipidomic data analysis).
Source data are provided with this paper.

Research involving human participants, their data, or biological material

Policy information about studies with human participants or human data. See also policy information about sex, gender (identity/presentation), and sexual orientation and race, ethnicity and racism.

Reporting on sex and gender	Donor sex is reported in Table S2 Donor Data. Sex and gender was not used in the study design and has had no influence on whether donor kidneys deemed unsuitable for transplantation were accepted, tested and analyzed in this study.
Reporting on race, ethnicity, or other socially relevant groupings	Information on race, ethnicity, or other socially relevant groupings were not collected nor relevant to this study.
Population characteristics	Human kidneys from deceased donors deemed unsuitable for transplantation were used in this study. Donor data can be found in Table S2.
Recruitment	A total of eight human kidneys deemed unsuitable for transplantation were included in this study. In compliance with Dutch law, research consent was obtained by the centralized donation organization in The Netherlands, Eurotransplant, before the start of organ retrieval for all human kidneys. This consent was obtained by an independent organ donation coordinator, with no relation to the research team.
Ethics oversight	Leiden University Medical Center received authorization from the Dutch government (BWBR0008974, 2555663-CZ/IZ/2562427) for kidney transplantation and associated research. Prior to organ retrieval for all human kidneys, donor research consent was obtained by Eurotransplant, the centralized donation organization in The Netherlands (BWBR008066, Art13). This consent was acquired by an independent organ donation coordinator unaffiliated to the research team.

Note that full information on the approval of the study protocol must also be provided in the manuscript.

Field-specific reporting

Please select the one below that is the best fit for your research. If you are not sure, read the appropriate sections before making your selection.

[x] Life sciences [ ] Behavioural & social sciences [ ] Ecological, evolutionary & environmental sciences

For a reference copy of the document with all sections, see nature.com/documents/nr-reporting-summary-flat.pdf

Life sciences study design

All studies must disclose on these points even when the disclosure is negative.

Sample size	No sample size calculation was performed in this study. We considered a sample size of 8 human kidneys suitable to provide proof-of-concept, based on recent organ perfusion literature.
Data exclusions	No data were excluded from the analysis.
Replication	Eight human kidneys deemed unsuitable for transplantation were perfused within the described platform and reproducibility of multi-day kidney preservation was assessed based on a collection of parameters, ranging from whole organ perfusion dynamics and function to single-cell-level metabolic preservation.
Randomization	Randomization was not performed in this study with 8 human kidneys deemed unsuitable for transplantation as we had a "single intervention group".
Blinding	Investigators were not blinded to data collection and analysis, as we had a "single intervention group"

Reporting for specific materials, systems and methods

We require information from authors about some types of materials, experimental systems and methods used in many studies. Here, indicate whether each material, system or method listed is relevant to your study. If you are not sure if a list item applies to your research, read the appropriate section before selecting a response.

Materials & experimental systems

n/a	Involved in the study
☐	☒ Antibodies
☒	☐ Eukaryotic cell lines
☒	☐ Palaeontology and archaeology
☐	☒ Animals and other organisms
☒	☐ Clinical data
☒	☐ Dual use research of concern
☒	☐ Plants

Methods

n/a	Involved in the study
☒	☐ ChIP-seq
☒	☐ Flow cytometry
☒	☐ MRI-based neuroimaging

Antibodies

Antibodies used

Lotus Tetragonolobus Lectin (LTL) (1:300; Vector laboratories, B1325);
Sheep Polyclonal IgG anti-NPHS1 (5 µg/mL; R&D systems, AF4269);
Mouse IgG2a anti-CDH1 (1:300; BD Biosciences, 610181);

Streptavidin anti Biotin AF488 (1:300; ThermoFisher, S-11223);
Donkey anti Sheep IgG AF568 (1:300; Invitrogen, A21099);
Goat anti Mouse IgG2a AF647 (1:300; ThermoFisher, A-21241);

NGAL Quantikine ELISA kit (R&D systems, DLCN20);
KIM-1 DuoSet ELISA kit (R&D systems, DY1750B);

Validation

Lotus Tetragonolobus Lectin (LTL) (Vector laboratories, B-1325; biotinylated). Source: Lectin. Sugar specificity: Fucose, Arabinose. Conjugate: Biotinylated. Applications: immunohistochemistry, immunocytochemistry, immunofluorescence, blotting applications, elispot, ELISAs and glycobiology. Validation statements, relevant citations, product datasheet and other information can be found from the manufacturer’s website: https://vectorlabs.com/products/biotinylated-lotus-tetragonolobus-lectin-ltl.
Cited in >200 references.

Anti-NPHS1 (R&D systems, AF4269). Source: Polyclonal Sheep IgG. Species reactivity: human. Applications: western blots and immunohistochemistry. Validation statements, relevant citations, product datasheet and other information can be found from the manufacturer’s website: https://www.rndsystems.com/products/human-nephrin-antibody_af4269.
Cited in 22 references of which 17 include human applications.

Anti-CDH1 (BD Biosciences, 610182). Source: Mouse IgG2a. Species reactivity: human (QC Testing), mouse, rat, dog (Tested in Development); Application: Western blot (Routinely Tested), Immunofluorescence, Immunohistochemistry, Immunoprecipitation (Tested During Development). Validation statements, relevant citations, product datasheet and other information can be found from the manufacturer’s website: https://www.bdbiosciences.com/en-us/products/reagents/microscopy-imaging-reagents/immunofluorescence-reagents/purified-mouse-anti-e-cadherin.610182.
Cited in 5 references during development.

Animals and other research organisms

Policy information about studies involving animals; ARRIVE guidelines recommended for reporting animal research, and Sex and Gender in Research

Laboratory animals	Male Yorkshire pigs (30-32 kg, 3-months-old).
Wild animals	This study did not include wild animals.
Reporting on sex	Given elaborate previous experience with kidney procurement and auto-transplantation in male pigs at the Toronto Organ Preservation Lab (TOPL), all pigs included in this study for the large animal experiments performed at the TOPL were male.
Field-collected samples	This study did not include field-collected samples.
Ethics oversight	Large animal experiments were performed at the Toronto Organ Preservation Lab (TOPL), following a previously established protocol for porcine kidney procurement and auto-transplantation. These experiments were carried out in accordance with the Canadian Council on Animal Care guidelines. Animal ethical approval was granted under an Animal User Protocol (AUP) issued by the University Healthcare Network Animal Care Committee (AUP number 3651).

Note that full information on the approval of the study protocol must also be provided in the manuscript.

Plants

Seed stocks	N/A
Novel plant genotypes	N/A
Authentication	N/A
